# Decreased Power but Preserved Bursting Features of Subthalamic Neuronal Signals in Advanced Parkinson's Patients under Controlled Desflurane Inhalation Anesthesia

**DOI:** 10.3389/fnins.2017.00701

**Published:** 2017-12-12

**Authors:** Sheng-Huang Lin, Hsin-Yi Lai, Yu-Chun Lo, Chin Chou, Yi-Ting Chou, Shih-Hung Yang, I Sun, Bo-Wei Chen, Ching-Fu Wang, Guan-Tze Liu, Fu-Shan Jaw, Shin-Yuan Chen, You-Yin Chen

**Affiliations:** ^1^Institute of Biomedical Engineering, National Taiwan University, Taipei, Taiwan; ^2^Department of Neurology, Tzu Chi General Hospital, Tzu Chi University, Hualien, Taiwan; ^3^Interdisciplinary Institute of Neuroscience and Technology, Qiushi Academy for Advanced Studies, Zhejiang University, Hangzhou, China; ^4^The Ph.D. Program for Neural Regenerative Medicine, College of Medical Science and Technology, Taipei Medical University, Taipei, Taiwan; ^5^Department of Biomedical Engineering, National Yang Ming University, Taipei, Taiwan; ^6^Department of Mechanical and Computer Aided Engineering, Feng Chia University, Taichung, Taiwan; ^7^Department of Life Sciences, Institute of Genome Sciences, National Yang Ming University, Taipei, China; ^8^Department of Medicine, National Yang Ming University, Taipei, Taiwan; ^9^Department of Neurosurgery, Tzu Chi General Hospital, Tzu Chi University, Hualien, Taiwan

**Keywords:** Parkinson's disease, subthalamic nucleus, deep brain stimulation, microelectrode recording, desflurane general anesthesia, low frequency oscillation

## Abstract

Deep brain stimulation (DBS) surgery of the subthalamic nucleus (STN) under general anesthesia (GA) had been used in Parkinson's disease (PD) patients who are unable tolerate awake surgery. The effect of anesthetics on intraoperative microelectrode recording (MER) remains unclear. Understanding the effect of anesthetics on MER is important in performing STN DBS surgery with general anesthesia. In this study, we retrospectively performed qualitive and quantitative analysis of STN MER in PD patients received STN DBS with controlled desflurane anesthesia or LA and compared their clinical outcome. From January 2005 to March 2006, 19 consecutive PD patients received bilateral STN DBS surgery in Hualien Tzu-Chi hospital under either desflurane GA (*n* = 10) or LA (*n* = 9). We used spike analysis (frequency and modified burst index [MBI]) and the Hilbert transform to obtain signal power measurements for background and spikes, and compared the characterizations of intraoperative microelectrode signals between the two groups. Additionally, STN firing pattern characteristics were determined using a combined approach based on the autocorrelogram and power spectral analysis, which was employed to investigate differences in the oscillatory activities between the groups. Clinical outcomes were assessed using the Unified Parkinson's Disease Rating Scale (UPDRS) before and after surgery. The results revealed burst firing was observed in both groups. The firing frequencies were greater in the LA group and MBI was comparable in both groups. Both the background and spikes were of significantly greater power in the LA group. The power spectra of the autocorrelograms were significantly higher in the GA group between 4 and 8 Hz. Clinical outcomes based on the UPDRS were comparable in both groups before and after DBS surgery. Under controlled light desflurane GA, burst features of the neuronal firing patterns are preserved in the STN, but power is reduced. Enhanced low-frequency (4–8 Hz) oscillations in the MERs for the GA group could be a characteristic signature of desflurane's effect on neurons in the STN.

## Introduction

Deep brain stimulation (DBS) surgery has been used for treating movement disorders for 30 years (Benabid et al., 1987). Bilateral subthalamic nuclei DBS (STN DBS) surgery has been the most effective treatment for advanced Parkinson's disease (PD) with motor complications, and intraoperative recordings of microelectrode signals have been used in STN DBS surgery for verification of distinct brain areas and precise localization at most DBS centers. To obtain clear STN signals, most STN DBS surgeries are performed under local anesthesia (LA) (Rezai et al., 2006). PD patients with severe anxiety, rigidity, and dystonia, or even respiratory difficulty because of a lack of medication are considered unable to tolerate awake DBS surgery (Hertel et al., 2006). These patients must undergo STN DBS surgery with either conscious sedation or general anesthesia (GA) (Bindu and Bithal, 2016). Major concerns about DBS surgery under GA are (1) the loss of some features in the microelectrode signal and (2) the inability to test stimulation.

A recent clinical meta-analysis has shown that about 10% of DBS surgeries for PD patients were under GA, explaining why most clinicians and DBS surgeons hesitate and doubt the feasibility of and clinical outcomes after surgery under GA (Krause et al., 2004; Hertel et al., 2006; Ho et al., 2017). For this reason, advanced PD patients who cannot endure awake surgery could lose their chance at DBS treatment. Though previous studies have demonstrated decreases in or the absence of microelectrode signals under certain forms of GA (Hutchison et al., 2003; Krause et al., 2004; Grant et al., 2015), several studies have attempted to preserve the signals by using different types of GA (Harries et al., 2012; Fluchere et al., 2014). Previously, we reported that STN neuronal signals could be identified under light desflurane GA, and the long-term effect was comparable to that of LA (Lin et al., 2008). We also reported that contralateral median nerve stimulation during recording could enhance the signals (Tsai et al., 2015). However, inhalation anesthetics can still affect the microelectrode signals and possibly hinder their performance during DBS surgery (Sanghera et al., 2003). A better understanding of desflurane's effect on STN neuronal firing could make microelectrode recording (MER) studies during STN DBS surgery with light inhalation anesthesia easier, more manageable, and less likely to be misjudged.

In this study, our objectives were to (1) compare the clinical outcomes between the LA and GA groups and (2) to systemically compare microelectrode signals from the STN under LA with those obtained under light inhalation GA. Our results provide (1) more evidence of the feasibility of STN DBS surgery under GA for advanced PD patients who cannot tolerate awake surgery and (2) in-depth information (characteristics, similarities, and differences between LA and GA) about the effects of inhalation anesthetia on STN neuronal signals for clinicians. We demonstrate that MER can be performed properly during STN DBS surgery under GA.

## Materials and methods

### Patient population

We conducted a retrospective off-line analysis of intraoperative microelectrode data recorded from 19 consecutive patients with PD who underwent bilateral STN DBS surgery between January 2005 and March 2006 in our hospital. Mean age was 56.7 years (range, 34–70 years), and the ratio of men to women was 2.8:1. The method of anesthesia during surgery was chosen by each patient after discussing the benefits and drawbacks of each with the DBS team. Patient consent for surgery and anesthesia was obtained from each patient before the procedure. Ten patients chose to receive desflurane anesthesia and were assigned to the GA group. The remainder chose LA and were assigned to the LA group. Mean ± (standard deviation, SD) age was 60.0 ± 8.1 years for the GA group and 53.0 ± 11.6 years for the LA group. Disease duration was 12.4 ± 5.5 years for the GA group and 9.2 ± 1.8 years for the LA group. Average Hoehn and Yahr (H&Y stage (clinical staging for PD) (Goetz et al., 2004) was 3.1 ± 0.6 for the GA group and 2.9 ± 0.2 for the LA group. Clinical outcomes were assessed using the Unified Parkinson's Disease Rating Scale (UPDRS) (Movement Disorder Society Task Force on Rating Scales for Parkinson's, 2003) before and 6 months after DBS surgery (Martínez-Martín et al., 1994).

This study was approved by the Institutional Review Board of Tzu-Chi General Hospital (IRB105-70-B). Taking MERs was a routine clinical procedure during STN DBS surgery. Because this study retrospectively analyzed MERs, the IRB agreed not to require informed consent from these patients.

### Surgery

#### Imaging and targeting

Before the neurosurgical procedure, MRI was performed on the head using a 1.5-tesla MR scanner (Signa Excite; GE Medical Systems, Milwaukee, WI, USA) to identify and locate the STN within the brain. Three MRI sequences were obtained: T_1_-weighted (T_1_W) axial images (repetition time [TR]: 26 ms; echo time [TE]: 6.9 ms; matrix size: 256 × 192; thickness: 0.7 mm), fast spin echo (FSE) T_2_-weighted (T_2_W) axial images (TR: 4,800 ms; TE: 95 ms; field of view (FOV): 24 cm; matrix size: 256 × 192; thickness: 2.0 mm) and coronal images (TR: 5,000 ms; TE: 102 ms; FOV: 20 cm; matrix size: 256 × 192; thickness: 3.0 mm). Data were acquired in contiguous slices. Through the picture archiving and communication system (PACS), brain MRI images were transferred to the neuronavigation workstation (VectorVision; Brainlab, Westchester, IL, USA). Targets and trajectories were planned based on the three sets of images using the VectorVision neuronavigation system. Tentative surgical target coordinates for the tip of the implantable electrode were set centrally on the lowest border of the STN using direct visualization on MRI images as previously described (Chen et al., 2006).

#### Stereotactic procedure

A Leksell stereotactic G skull frame (Elekta Instrument, Atlanta, GA, USA) was used for the stereotactic procedure. With the head ring secured on a Mayfield adaptor, the patient laid on the operative table in a semi-fowler position. Target coordinates were applied to the stereotactic frame and the working stage. After MERs were obtained, quadripolar DBS electrodes (Model 3389; Medtronic, Englewood, CO, USA) were implanted on the appropriate trajectory.

### Grouping for anesthetic management during DBS lead implantation

#### Controlled light inhalation anesthesia

Ten patients underwent light desflurane GA via endotracheal intubation. Anesthetic induction for intubation was achieved using lidocaine (0.5–1.5 mg/kg), fentanyl (1–2 μg/kg), propofol (1–2.5 mg/kg), and a muscle relaxant (rocuronium at 0.6–1.5 mg/kg or cisatracurium at 0.15–0.2 mg/kg). The surgical course was maintained using desflurane to sustain the minimal alveolar concentration (MAC) at 1. During MER, desflurane was decreased to sustain the MAC at 0.6–0.7. At this level, patients would not experience the cough reflex or changes in heart rate or blood pressure. MERs were collected without test stimulation.

#### Local anesthesia

Nine patients underwent local scalp anesthesia using lidocaine and were awake during collection of the MER and electrode placement. At the beginning (during craniotomy) and at the end of the operation, propofol and fentanyl were used for sedation and pain control.

### MER data collection

The available MER data comprised the intraoperative MERs collected during these 19 surgeries. Stereotactic intraoperative fluoroscopy was used to record the position of the microelectrode tip, which served as a reference for final DBS lead implantation. Immediately after implantation, the position of each permanent electrode was verified using computed tomography (CT) images co-registered with presurgical MRI images.

The Leadpoint recording system (Medtronic, Englewood, CO, USA) was used for collecting MERs; data were filtered using a 500-Hz high-pass filter and 5-kHz low-pass filter and were sampled at 24 kHz, then stored for off-line processing. Each high-impedance (0.8–1.5 MΩ) tungsten microelectrode was 200 mm long and had a 10-mm bare microtip 10–40 μm in diameter (FHC Inc., Bowdoin, ME, USA). Because of their high impedance, they can isolate the activity of a single neural unit. Spikes are generated by neurons located within approximately 150 μm from the microelectrode tip (Buzsaki, 2004; Moffitt and McIntyre, 2005), and thus, they are useful for precisely defining the properties of individual neurons within the STN. Microelectrodes were mounted on a microTargeting® microdrive (FHC Inc., Bowdoin, ME, USA), which was used to direct the microelectrode into the STN.

A typical trajectory was used to reach the STN; the microelectrode passed through the thalamus, zona incerta, STN, and the underlying substantia nigra reticulata (SNr). Two parallel trajectories 2 mm apart were used for each hemisphere. The mean microelectrode trajectory during DBS surgery was 3.2 ± 1.6 in the GA group and 3.8 ± 1.2 in the LA group. The trajectories of the two groups were not significantly different. As the microelectrode traversed the STN, a 10-s MER was collected with a sampling range (in depth space) of 0.2 mm along the full length of the STN. This recording was systematically assessed for movement-related cell discharge during passive movement of the contralateral upper or lower extremity (Theodosopoulos et al., 2003). MER signals recorded along the tracks were selected for off-line analysis. We selected MER data corresponding to the middle 3-mm section of the STN for further analysis. Fifteen STN-MER fragments were selected for each track; therefore, thirty STN-MER fragments were obtained from each patient. For the GA and LA groups, analyses were performed for 300 and 270 STN-MER fragments, respectively. Spike sorting results showed a total of 142 neurons in the GA group and 138 in the LA group (see Table S1 in the Supplement Materials Note 1). The patterns of spike clusters were similar per MER fragment (0.47 ± 0.07 neurons for the GA group vs. 0.51 ± 0.06 neurons for the LA group).

### Estimation for mean squared values of Hilbert transformed MER spike and background

The MER signal consisted of background noise from the recording equipment, biological background noise, and spikes (extracellular action potentials) associated with individual neurons close to the MER of most nearby neuronal units. Several studies report that background noise levels and spike firing patterns constitute good features for determining implantation depth and discrimination between target sites (internal globus pallidus (GPi), STN, and Vim nucleus of thalamus).

We compared MER data acquired under GA with that acquired under LA. A Hilbert transform was applied to determine the effects of the anesthetic agent on the microelectrode signal. The absolute amplitude envelope of the signal was computed via the Hilbert transform, which maximized the discrimination between spikes and background noise, and it provided a sparser representation (Dolan et al., 2009). The Hilbert transform of the MER signal *x*(*t*)is defined as:

(1) $$\begin{array}{l} H\left(x\left(t\right)\right)=\frac{1}{\pi }p.v.\int_{-\infty }^{\infty }\frac{x\left(\tau \right)}{\pi \left(t-\tau \right)}d\tau \end{array}$$

where *p*.*v*. is the Cauchy principal value.

Then, an analytical signal, *y*(*t*), was constructed using the MER signal *x*(*t*) as the real component and *H*(*x*(*t*)) as the imaginary component:

(2) $$\begin{array}{l} y\left(t\right)=x\left(t\right)+jH\left(x\left(t\right)\right) \end{array}$$

According to the Hilbert transform separation algorithm (Potamianos and Maragos, 1994), the MER amplitude envelope, |*B*(*t*)|, can be estimated as:

(3) $$\begin{array}{l} |B\left(t\right)|=\sqrt{x^{2}\left(t\right)+H^{2}\left(x\left(t\right)\right)} \end{array}$$

The STN-MER signals were processed using the Hilbert transform, and energy factors were achieved based on the signal envelope. This transformation returned an almost perfect Rayleigh distribution for pure band-limited white Gaussian noise, allowing for a clear separation between a lower (background noise) and a higher (spikes) mode (Dolan et al., 2009). Because power is proportional to the mean-square value of some quantity (such as the square of current or voltage in an electrical circuit), the mean-square value of any quantity has become known as the power of that quantity. We used the mean-square value (power) of MER amplitude envelope as a convenient measure of the strength of an individual spike segment ($P_{i}^{spk}$) and a background segment ($P_{j}^{bg}$). These were computed from the 10-s MER envelope as:

(4) $$\begin{array}{l} P_{i}^{spk}=\frac{1}{T_{i}^{spk}}\;\int \;{\left[B_{i}^{spk}\left(t\right)\right]}^{2}dt \end{array}$$

(5) $$\begin{array}{l} P_{j}^{bg}=\frac{1}{T_{j}^{bg}}\;\int \;{\left[B_{j}^{bg}\left(t\right)\right]}^{2}dt \end{array}$$

where $T_{i}^{spk}$is the time width of the *i*^*th*^ peak of the squared MER envelope, corresponding to the *i*^*th*^ spike of the MER signal, which is the distance between start and end points at 5% of peak height. $T_{j}^{bg}$is the time interval between successive peaks of the squared MER envelope corresponding to the *j*^*th*^ segment of the MER background noise.

Therefore, the average power across all spike segments, $\bar{P^{spk}}$, and that for all background segments, $\bar{P^{bg}}$, in the 10-s MER signal can be respectively expressed as:

(6) $$\begin{array}{l} \bar{P^{spk}}=\;\frac{\sum_{i=1}^{M}P_{i}^{spk}}{M} \end{array}$$

(7) $$\begin{array}{l} \bar{P^{bg}}=\frac{\sum_{j=1}^{N}P_{j}^{bg}}{N} \end{array}$$

where *M* and *N* are the number of spike and background power observations during the 10-s MER.

### Extraction of spike timing features

The prominent spike firing patterns found in STN MERs reportedly include tonic irregular single-spike activity, burst-firing activity, and neuronal oscillations with non-stationary properties (Favre et al., 1999; Bingmer et al., 2011; Tsai et al., 2015). To distinguish these firing patterns in response to GA or LA, power spectral analyses of autocorrelograms were separately calculated from the spike trains during the burst periods and the regularly-spiking periods. The modified burst index (MBI) and firing rate (FR) were also extracted as spike timing features. In these cases, signals that exceeded a simple amplitude threshold were identified as spikes. Applying a noise level estimation to the MER signal is a critical first step in this procedure. The threshold value, *Th*, was set at 4 times the estimated noise level:

(8) $$\begin{array}{l} Th=4\sigma_{n} \end{array}$$

where σ_*n*_ is the estimated noise level. The conventional method for estimating noise level is to compute the root-mean-square (RMS) of the overall raw MER signal (Dolan et al., 2009). However, the main disadvantage of this method is that the RMS is quite sensitive to higher-amplitude spikes (outliers) or a higher frequency in spike firing (mean variation) (Donoho and Johnstone, 1994; Quiroga et al., 2004). A better method for estimating background noise, the median method (Dolan et al., 2009), was used to calculate the threshold from the noise properties of the MER signal. For a Gaussian-distributed signal, the median of the absolute value of the raw MER signal and its standard deviation are proportional. By first taking the median of the absolute value of the raw signal and then dividing by a constant, an estimate of the background noise level is obtained. The parameter σ_*n*_ is set to:

(9) $$\begin{array}{l} \sigma_{n}=median\;\left\{\frac{\left|X\right|}{0.6745}\right\} \end{array}$$

where *X* = *x*_1_, *x*_2_, *x*_3_, …., *x*_*N*_ is the digitized MER data (including spikes and background signals), and *N* is the number of samples (24 kHz × 10 s). By estimating the median of *X*, the number of outliers and the mean variation of the distribution are reduced (Donoho and Johnstone, 1994; Quiroga et al., 2004). Therefore, spikes in our digitized MER data were first detected and then imported into our unsupervised spike sorting software (Hsin-Yi et al., 2011) for discrimination of single populations of action potentials by principal component analysis (PCA). Spike times and inter-spike intervals (ISIs) were determined simultaneously. The ISIs were used to evaluate stationarity of discharge, to calculate distribution parameters, to construct autocorrelograms, and to distinguish between intermittent bursting discharges and oscillatory discharges. To detect and test the significance of oscillation frequencies, spectral techniques were employed (Favre et al., 1999; Bingmer et al., 2011; Tsai et al., 2015). We also calculated MBI and FR based on the detected spikes and ISI spike timing features. The MBI was defined as the ratio of the number of ISIs shorter than 10 ms to the number of ISIs longer than 10 ms (Favre et al., 1999; Bingmer et al., 2011; Tsai et al., 2015). The FR was defined as the total number of detected spikes over a 1-s period. All data analyses were post-processed using MATLAB (R12; Mathworks Inc., Natick, MA, USA).

### Statistical analysis

All data were expressed as the means ± standard deviations (SD). The Mann-Whitney U-test was used for comparisons between groups. To assess clinical outcomes, comparisons of the post-operative levodopa equivalent daily dose and UPDRS (total and part III) scores before and 6 months after STN-DBS surgery were made using the Wilcoxon signed-rank test. Significance was recognized when *p* < 0.05.

## Results

### Comparisons of clinical outcomes: LA vs. GA

Table 1 showed the clinical outcomes for the two groups. Post-DBS operative levodopa equivalent daily dose (LEDD) at 6-month was significantly lower than the pre-operative LEDDs for both groups (*p* < 0.01, Wilcoxon signed-rank test). A follow-up examination of GA and LA groups at 6 months after surgery revealed that STN-DBS significantly improved UPDRS (total and part III) scores collected in the ON (drug on and DBS on) states compared with those collected in the OFF (drug off and DBS off) state (*p* < 0.01, Wilcoxon signed-rank test). However, post-DBS operative clinical outcomes were not significantly different between the groups (*p* > 0.05, Mann-Whitney U-test).

**Table 1:** Comparisons of clinical outcomes.

	**GA group**	**LA group**	***p***
**PRE-DBS**			
LEDD	1,036.9 ± 328.1 (mg)	862.9 ± 461.1 (mg)	0.35
UPDRS (Total/Part III) OFF^§^	90.8 ± 25.8/54.4 ± 14.9	75.6 ± 30.4/46.0 ± 16.8	0.25/0.26
UPDRS (Total/Part III) ON^‡^	52.5 ± 12.3/30.8 ± 7.7	43.3 ± 10.6/24.7 ± 8.3	0.10/0.12
**POST-DBS (HALF YEAR)**			
LEDD	408.8 ± 156.5 (mg)^¶¶^	404.7 ± 244.6 (mg)^¶¶^	0.97
UPDRS (Total/Part III) OFF^*^	89.8 ± 26.5/54.2 ± 15.0	74.9 ± 27.4/45.7 ± 15.8	0.26/0.26
UPDRS (Total/Part III) ON^#^	37.1 ± 12.0^⊤⊤^/23.5 ± 5.8^⊤⊤^	33.7 ± 9.7^⊤⊤^/22.1 ± 6.7^⊤⊤^	0.52/0.64

*GA, general anesthesia; LA, local anesthesia; LEDD, levodopa equivalent daily dose; UPDRS, Unified Parkinson's Disease Rating Scale (Rating scales for evaluating PD in different domains)*.

§ *(drug OFF)*.

‡ *(drug ON)*.

* *(drug and DBS OFF)*.

# *(drug and DBS ON)*.

*Data are given as means ± SD*.

⊤⊤ *p < 0.01 (Wilcoxon signed-rank test) as compared with post-operative UPDRS (Total/Part III) in OFF state at 6 months*.

¶¶ *p < 0.01 (Wilcoxon signed-rank test) as compared with preoperative LEDD*.

### Mer comparisons: LA vs. GA

**Figure 1:**
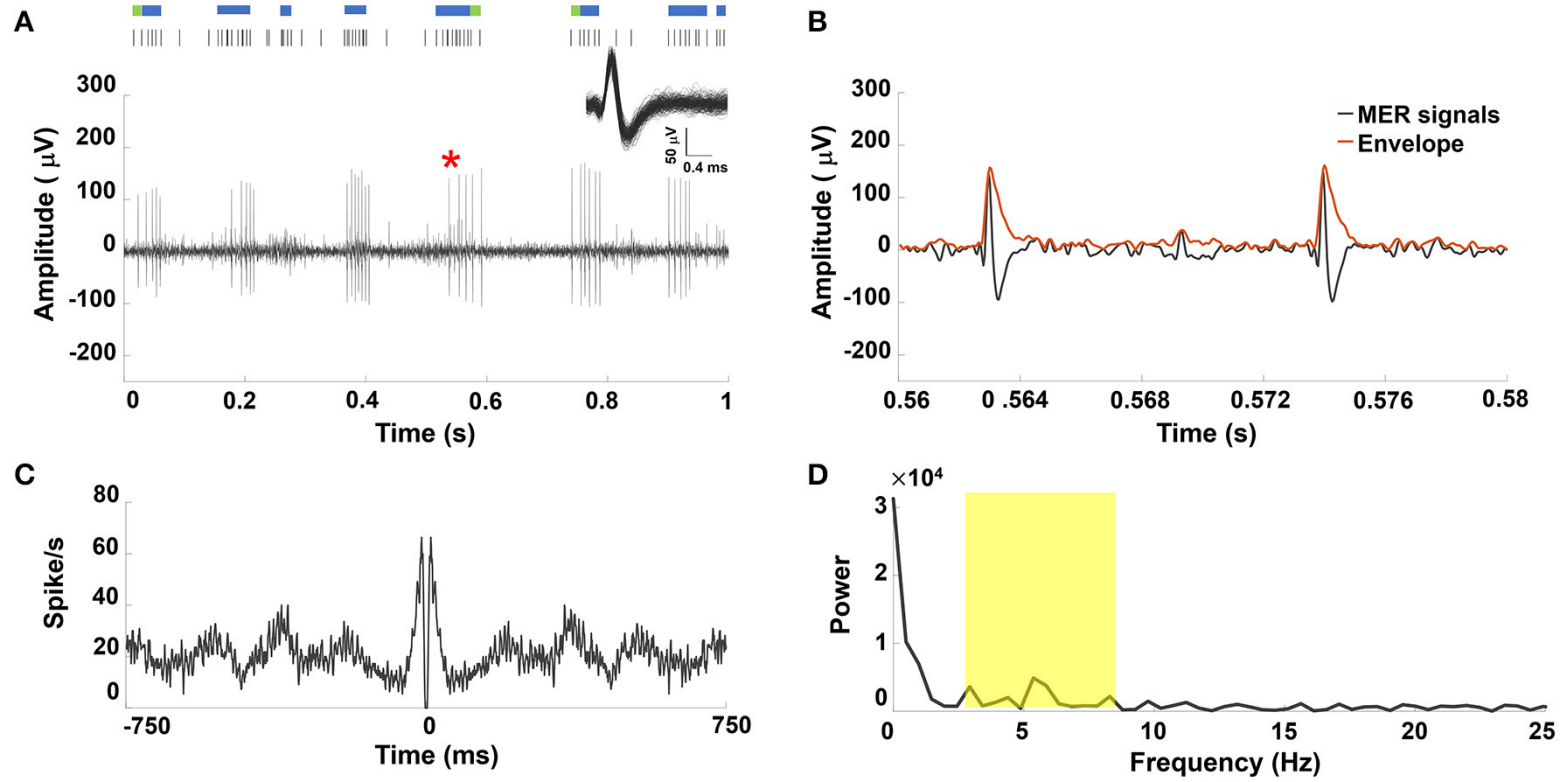
**(A)** Representative example of STN MER data under GA. Single unit neuron was isolated in 3D space based on characteristics of spike waveforms using principal component analysis (PCA). The superposed spikes exhibited the same shape, which confirmed they belong to the same neuron in STN. A raw MER signal with raster plot above indicating times of burst firing (blue rods) and irregular activity (green rods), with bursts in single unit discharge occurring in a periodic manner. Within the burst, the accelerating firing rate was found during the burst accompanied by progressively decrementing action potentials. **(B)** The envelope waveform computed from Hilbert transformed MER signal within the marked area (^*^) of **(A). (C)** The autocorrelogram (time base of 750 ms and bin width is 1 ms) of the 10-s MER raw data, **(D)** the power spectrum of the autocorrelogram between 0 and 25 Hz for the unit shown in **(A)**. There were significant increases in the power spectrum between 4–8 Hz (yellow highlighted area).

An example of a sorted single STN neuron with oscillatory discharge under GA and the corresponding raster plot showing spike trains that formed a perfect periodic firing pattern was shown in Figure 1A. Figure 1B showed the enlarged MER signal from the marked area with a red star in Figure 1A, and the corresponding envelope was obtained using the absolute value for the Hilbert-transformed MER signal. The autocorrelogram (Figure 1C) showed periodic peaks of sinusoidal density with recurrent events. Figure 1D showed the frequency spectrum of the autocorrelogram with a distinct peak near 6.3 Hz. Figure S1 in the Supplementary Materials Note 2 shows similar results from other PD patients under GA.

**Figure 2:**
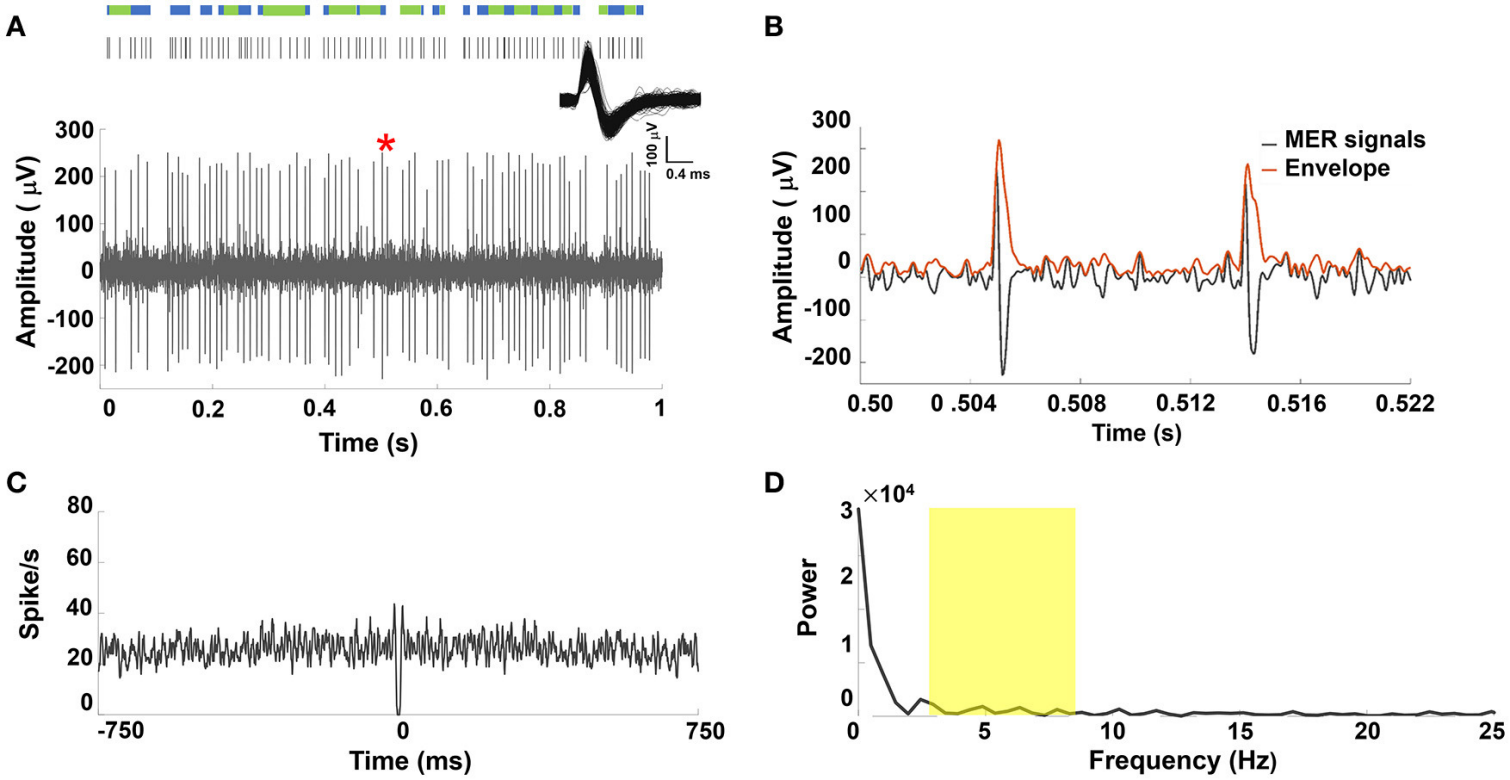
**(A)** Representative example of STN MER data under LA. Single unit neuron was isolated in 3D space based on characteristics of spike waveforms using principal component analysis (PCA). The superposed spikes exhibited the same shape, which confirmed they belong to the same neuron in STN. A raw MER signal with raster plot above indicating times of burst firing (blue rods) and irregular activity (green rods). However, STN discharge rate showed non-oscillatory burst firing under LA. **(B)** The envelope waveform computed from Hilbert transformed MER signal within the marked area (^*^) of **(A)**. **(C)** The autocorrelogram (time base of 750 ms and bin width is 1 ms) of the 10-s MER raw data, **(D)** the power spectrum of the autocorrelogram between 0 and 25 Hz for the unit shown in **(A)**. There were no significant increases in the power spectrum between 4–8 Hz (yellow highlighted area).

A representative MER signal under LA and its corresponding raster plot showing mixed irregular tonic and non-periodical burst patterns from a sorted single STN unit discharge was illustrated in Figure 2A. Figure 2B showed the section of the MER marked in Figure 2A and the corresponding amplitude envelope using the Hilbert transformation. Greater spike amplitudes were seen in the LA group (example in Figure 2B) than in the GA group (example in Figure 1B). Irregular and non-periodical burst activities were characterized by a flat autocorrelogram (see Figure 2C) and were considered to have no significant oscillatory component. Therefore, the power spectrum of the autocorrelogram showed no significant peaks between 4 and 8 Hz (Figure 2D). Figure S2 in the Supplementary Materials Note 2 showed similar results for other PD patients under LA.

**Figure 3:**
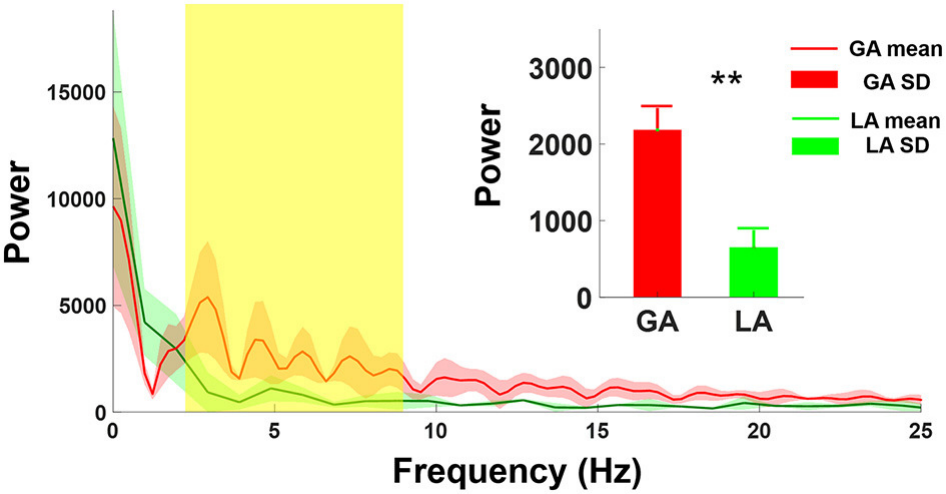
Comparison of power spectrum of autocorrelogram between GA and LA groups. The red and green lines are the mean spectrum of the MER autocorrelogram under GA and LA groups, respectively. The magnitude of GA power spectrum (2,183 ± 465) showed significant increases at the frequency range from 4 to 8 Hz as compared with those under LA (649 ± 203). ^**^indicates significant increases in the magnitude of spectrum of autocorrelogram over 4–8 Hz with *p* < 0.01 compared with the GA group, analyzed by the Wilcoxon two-sample test (Mean ± SD).

Figure 3 showed the comparison of the power spectrum distributions between GA and LA groups. The mean amplitude of power spectrum GA-MER autocorrelogram presented significantly increasing between 4-8 Hz as compared with those of LA. The magnitude of power spectrum between 4-8 Hz in GA group was 2183 ± 465 (a.u.), and LA group was 649 ± 203 (a.u.) which was significantly smaller than GA (^**^p < *0.01*, Mann-Whitney U-test).

The average background power, average spike power, and FR were significantly smaller for the GA group than the LA group (*p* < 0.01 for all, Mann-Whitney U-test). However, mean MBI was not significantly different (0.967 ± 0.438 vs. 0.987 ± 0.120, respectively, Mann-Whitney U-test; see Table 2).

**Table 2:** Comparisons of MER characteristics.

	**GA group**	**LA group**	***p***
Average background power	87.848 ± 37.881	405.925 ± 101.322	<0.01
Average spike power	544.848 ± 185.633	1, 783.154 ± 648.374	<0.01
MBI	0.967 ± 0.438	0.987 ± 0.120	0.890
FR (spike/s)	35.394 ± 7.025	53.031 ± 7.598	<0.01

*Data are expressed as mean ± standard deviation (SD). GA, general anesthesia; LA, local anesthesia; MBI, modified burst index; FR, firing frequency*.

## Discussion

The clinical outcomes for our patients were comparable to those found in previous studies (Hertel et al., 2006; Harries et al., 2012), and the provide evidence that the effect of STN DBS surgery on PD patients is equivalent regardless of anesthesia method (LA or controlled desflurane inhalation). A recent meta-analysis shows a lower complication rate in asleep DBS surgery than awake surgery (Ho et al., 2017). It is reasonable that STN DBS surgery under GA should be considered in PD patients with poor tolerance to awake surgery or comorbidities leading to additional complications. The population of DBS surgery candidates will be larger if surgery is not limited to LA only.

Most STN DBS surgeries have been performed under LA because it is thought that patients must be kept awake to get a clear microelectrode signal and to perform a test stimulation after lead implantation. The necessity of obtaining an MER during STN DBS surgery where the STN and its subdivisions can be accurately localized remains under debate. Several researchers report substantial improvements in accurate targeting for MRI-guided STN DBS surgery without obtaining MERs (Patel et al., 2003; Foltynie et al., 2011; Burchiel et al., 2013; Aviles-Olmos et al., 2014). These studies required either specific MRI sequences with fusion or intraoperative computed tomography, which might not be available in many hospitals performing STN DBS surgery; however, only one using intraoperative computed tomography performed STN-DBS surgery under GA (Burchiel et al., 2013). These results can be explained by the surgical time saved when directed MRI targeting is used without obtaining MERs, but patients must still remain awake. A recent meta-analysis comparing awake vs. asleep DBS surgery found that MERs were collected from half of the asleep surgeries. The author emphasized that collection of MERs seemed beneficial in accurately defining the DBS lead target under GA (Ho et al., 2017).

Because GA decreases stress and patient intolerance of during DBS surgery, reports of STN DBS surgery under GA have gradually increased in recent years (Hertel et al., 2006; Lin et al., 2008; Braun et al., 2011; Harries et al., 2012; Lettieri et al., 2012; Fluchere et al., 2014). The anesthetic agent used in STN DBS surgery can affect the neuronal signals in seen in MERs. Most studies of STN DBS surgery under GA used propofol and remifentanyl for anesthetia (Hertel et al., 2006; Lefaucheur et al., 2008; Braun et al., 2011). Propofol significantly suppresses the activity of pallidal neurons, causing decreased firing frequency and long pauses (Hutchison et al., 2003; Krause et al., 2004). One study tested the effect of propofol and remifentanyl on STN neurons; results showed only subtle changes in signals under either propofol or remifentanyl (Maciver et al., 2011). These controversial results might reflect the importance of dose and timing for anesthetic agents. Both influence MER signal presentation. Though desflurane has reportedly decreased discharge rates in the globus pallidus of PD patients, similar to propofol (Sanghera et al., 2003), volatile anesthetics (desflurane and seveflurane) have been used in two studies finding successful presentation of microelectrode signals from the STN (Lin et al., 2008; Fluchere et al., 2014). Both reports emphasize that controlling the anesthetic dose concentration is crucial to finding microelectrode signals successfully. Our results provided evidence that confirming the appearance and identification of STN microelectrode signals under controlled desflurance anesthesia.

Prior studies report that prominent spike firing patterns in the basal ganglia include tonic irregular single-spike activity, burst-firing activity, and neuronal oscillations with non-stationary properties (Favre et al., 1999; Bingmer et al., 2011). The burst pattern is thought to be a feature of STN neuronal signals during STN DBS surgery (Hutchison et al., 2003; Bour et al., 2010). One previous report compared the neuronal activities of the STN in PD and essential tremor patients, finding that STN neurons in PD patients exhibited more burst-like activity compared with essential tremor patients (Steigerwald et al., 2008). The bursting STN neuronal firing pattern has been described in several DBS studies under GA (Hertel et al., 2006; Lin et al., 2008; Harries et al., 2012; Lettieri et al., 2012). Interestingly, oscillatory modulation and bursting patterns in the STN neuronal signals have been abundant in PD patients and are considered pathophysiologically abnormal (Hutchison et al., 1998; Dovzhenok and Rubchinsky, 2012; Lobb, 2014). Two studies specifically describe characteristic mixed burst and non-burst (tonic or irregular) patterns under LA and a periodic burst pattern under GA (Moll et al., 2013, 2014; Lee et al., 2016). Figure 1 shows periodic burst spikes intermixed with sparse irregular (non-burst) spikes, observed in the GA group. On the other hand, the non-oscillatory burst firing with abundant irregular spikes is seen in LA patients (Figure 2). These findings are compatible previous observations, and we speculate that normal non-bursting STN neuronal signals are more vulnerable than abnormal bursting STN firing under desflurane anesthesia. Therefore, the periodic (oscillatory) burst pattern was enhanced by desflurane.

Results show that low-frequency (4–8 Hz) oscillation was enhanced by desflurane anesthesia. Studies of the effects of anesthetics in altering oscillation of STN neurons are sparse. Anesthetics used for general anesthesia has been shown to increase the power of low-frequency oscillation on electroencephalograms (Brown et al., 2010). While propofol has been characterized as an anesthetic that creates higher amplitude alpha oscillations on electroencephalograms (Purdon et al., 2013), volatile anesthetics lead to another peak in theta oscillation (Akeju et al., 2014). In a cervical dystonia DBS study, GA with propofol enhanced the theta band power in the globus pallidus compared with LA (Moll et al., 2014). An *in vitro* study demonstrated low-frequency oscillations between the STN and the external globus pallidus (Plenz and Kital, 1999), indicating that pallido-subthalamic networks play the necessary roles in oscillatory modulation in the STN. Our analysis of the power of oscillation from STN neurons confirm that desflurane changes the original oscillation pattern by creating higher theta band power. We speculate that this oscillatory modulation is partially from desflurane's effect on the external globus pallidus (Sanghera et al., 2003). Overall, this finding could suggest theta oscillation serves as a characteristic signature for volatile anesthetics on the STN.

In this study, we specifically computed the envelopes of background activities in MERs collected under LA and GA, and results show a decrease in power for the background activities in the GA group. Previous studies show that under GA, STN activity might be characterized by a loss of background widening (Hertel et al., 2006; Lettieri et al., 2012) or preserved background widening, as we observed under LA (Harries et al., 2012). Our data support the hypothesis that lower background power can result in decreased background widening under GA. Our results show that a decreased enveloped power was found not only in the background activities but also in spike activity under GA with light desflurane. The simultaneous effects of anesthesia on spike and background activities paradoxically preserve the bursting feature of STN neurons under LA. However, we did not explore the mechanism of desflurane's effect on the power (energy) of STN neuron activity.

The cerebral metabolic rate of oxygen (CMRO_2_) might reflect the energy cost of neuronal activity, which includes post-synaptic potentials and action potentials (Murta et al., 2015). Marked increases in the cerebral blood flow-to-CMRO_2_ ratio during surgery under volatile anesthetics have been reported in humans (Kuroda et al., 1996). Other researchers have found that sevoflurane maintained stable cerebral blood flow in stroke patients during surgery (Kitaguchi et al., 1993). Therefore, volatile anesthetics could decrease CMRO_2_ markedly during surgery. Inhaled anesthetics such as desflurane block presynaptic voltage-gated sodium channels, which could decrease glutamate release by inhibiting nerve terminal depolarization (Hemmings, 2009). Decreases in presynaptic excitatory neurotransmitters such as glutamate could decrease the membrane potential at the soma and proximal dendrites of the post-synaptic neuron. Extracellular spikes are actually the summation of integrated membrane potentials from the soma and proximal dendrites (Holt and Koch, 1999), which possibly contribute to the energy cost of neuronal signals. Our results suggest a plausible explanation: that desflurane decreases signal power by decreasing CMRO_2_.

The MBI has been used for quantitative evaluations of neuron bursting. Prior studies have analyzed how the various STN bursts in firing rate (MBI) are affected by the anesthetic method. Overall, the effect of GA on burst index remains controversial. One report with dexmedetomidine sedation found a decreased burst index in the dorsal, but not in the ventral STN (Krishna et al., 2015). On the contrary, an increased burst index was noted with propofol and fentanyl in another study (Park et al., 2015). Burst indices similar to those we found under LA and GA were reported in a study using ketamine anesthesia (Lettieri et al., 2012). It is possible that the diverse effects on the burst index is caused primarily by the anesthetic used. Similar MBIs in the LA and GA groups might indicate how STN firing patterns can be observed in DBS surgery under both conditions.

Our data show lower firing frequencies in STN neurons under GA (24.0 ± 10.4) than under LA (53.031 ± 7.598). This finding is compatible with our previous findings (Lin et al., 2008) and one other study that used ketamine anesthesia (Lettieri et al., 2012). Volatile anesthetics have been proven to inhibit presynaptic sodium channels and are critical for neuronal action potentials (Herold and Hemmings, 2012), which could explain the decreased neuronal FR we found. The influence of FR on STN neurons under various GA conditions could be anesthetic-dependent (Hertel et al., 2006; Lin et al., 2008; Maciver et al., 2011; Lettieri et al., 2012; Kim et al., 2014). Even the inhaled anesthetics could have different effects on FR because of their differences in efficacy in sodium channel inhibition (OuYang et al., 2009). We expect that the degree of change in STN FR is highly dependent on anesthetic and dosage.

These primary results revealed no significant difference between GA and LA in MBIs of STN single-unit activity. However, differences in spike characteristics and firing patterns were seen. General anesthesia using controlled light desflurane anesthesia could preserve bursting firing patterns through decreases in both background and spike signal powers. These findings contribute valuable insights into the effects of various anesthetic methods on STN single-unit activity during DBS surgery. For advanced PD patients who are unable to tolerate LA, controlled light desflurane anesthesia is a good alternative in STN DBS surgery.

## Author contributions

S-HL, H-YL, S-YC, and Y-YC designed the project, organized the entire research. S-HL., Y-CL, and F-SJ conceived the experiments. S-HL, Y-CL, and S-YC conducted the experiments. CC, Y-CL, Y-TC, S-HY, IS, B-WC, C-FW, and G-TL analyzed the results. S-HL, H-YL, and Y-YC wrote the manuscript. All authors discussed the results and reviewed on the manuscript.

### Conflict of interest statement

The authors declare that the research was conducted in the absence of any commercial or financial relationships that could be construed as a potential conflict of interest.
