# Supplementary material for: Decreased Power but Preserved Bursting Features of Subthalamic Neuronal Signals in Advanced Parkinson's Patients under Controlled Desflurane Inhalation Anesthesia
Source: Front Neurosci. 2017 Dec 12;11:701. doi: 10.3389/fnins.2017.00701 (PMC5733027; doi:10.3389/fnins.2017.00701)
Supplement: Supplementary file 1 [file Table1.DOCX]

Supplementary Material

Decreased Power but Preserved Bursting Features of Subthalamic Neuronal Signals in Advanced Parkinson’s Patients under Controlled Desflurane Inhalation Anesthesia

Sheng-Huang Lin^1,2^, Hsin-Yi Lai^3^, Yu-Chun Lo^4^, Chin Chou^5^, Yi-Ting Chou^5^, Shih-Hung Yang^6^, I Sun^7^, Bo-Wei Chen^5^, Ching-Fu Wang^5^, Guan-Tze Liu^8^, Fu-Shan Jaw^1^, Shin-Yuan Chen^9,*^, and You-Yin Chen^4,5,^*

^1^Institute of Biomedical Engineering, National Taiwan University, No.1, Sec. 1, Jen-Ai Rd., Taipei 10051, Taiwan, R.O.C.

^2^Department of Neurology, Tzu Chi General Hospital, Tzu Chi University, No. 707, Sec. 3, Chung, Yang Rd., Hualien 97002, Taiwan, R.O.C.

^3^Interdisciplinary Institute of Neuroscience and Technology (ZIINT), Qiushi Academy for Advanced Studies (QAAS), Zhejiang University, No.268, Kaixuan Rd., Hangzhou, Zhejiang310029, China

^4^The Ph.D. Program for Neural Regenerative Medicine, College of Medical Science and Technology, Taipei Medical University, No. 250 Wu-Hsing St., Taipei 11050, Taiwan, R.O.C.

^5^Department of Biomedical Engineering, National Yang Ming University, No.155, Sec.2, Linong St., Taipei, Taiwan 11221, R.O.C.

^6^Department of Mechanical and Computer Aided Engineering, Feng Chia University, No. 100, Wenhwa Rd., Taichung 407, Taiwan, R.O.C.

^7^Department of Life Sciences and Institute of Genome Sciences, National Yang Ming University, No.155, Sec.2, Linong St., Taipei, Taiwan 11221, R.O.C.

^8^Department of Medicine, National Yang Ming University, No.155, Sec.2, Linong St., Taipei, Taiwan 11221, R.O.C.

^9^Department of Neurosurgery, Tzu Chi General Hospital, Tzu Chi University, No. 707, Sec. 3, Chung, Yang Rd., Hualien 97002, Taiwan, R.O.C.

^*^Correspondence should be addressed to either of the following:

Dr. Shin-Yuan Chen, Department of Neurosurgery, Tzu Chi General Hospital, Tzu Chi University, No. 707, Sec. 3, Chung, Yang Rd., Hualien 97002, Taiwan, R.O.C.

Email: [william.sychen@msa.hinet.net](mailto:william.sychen@msa.hinet.net)

Dr. You-Yin Chen, Department of Biomedical Engineering, National Yang Ming University, No.155, Sec.2, Linong St., Taipei, Taiwan 11221, R.O.C.

E-mail: [irradiance@so-net.net.tw](mailto:irradiance@so-net.net.tw)

***Note 1. Patients’ demographic data, number of MER fragments and sorted neurons***

| Anesthetic technique | Patient number | Age  year/gender | Disease  Duration  (year) | H&Y staging  (preoperative) | Numbers of sorted neurons | Average neurons per fragment |
| --- | --- | --- | --- | --- | --- | --- |
| GA | 1 | 65/M | 10 | 3 | 11 | 0.37 |
|  | 2 | 64/M | 15 | 3 | 12 | 0.4 |
|  | 3 | 70/M | 12 | 3 | 15 | 0.5 |
|  | 4 | 66/W | 26 | 4 | 17 | 0.57 |
|  | 5 | 53/M | 6 | 3 | 18 | 0.6 |
|  | 6 | 63/M | 14 | 3 | 16 | 0.53 |
|  | 7 | 43/M | 11 | 4 | 13 | 0.43 |
|  | 8 | 66/M | 14 | 2.5 | 13 | 0.43 |
|  | 9 | 52/M | 5 | 2 | 15 | 0.5 |
|  | 10 | 54/M | 11 | 3 | 12 | 0.4 |
| LA | 1 | 70/W | 8 | 3 | 15 | 0.5 |
|  | 2 | 53/M | 9 | 3 | 19 | 0.63 |
|  | 3 | 65/M | 10 | 2.5 | 16 | 0.53 |
|  | 4 | 63/M | 10 | 2.5 | 14 | 0.47 |
|  | 5 | 59/M | 12 | 3 | 13 | 0.43 |
|  | 6 | 50/M | 10 | 3 | 17 | 0.57 |
|  | 7 | 41/M | 7 | 3 | 15 | 0.5 |
|  | 8 | 42/M | 11 | 3 | 14 | 0.47 |
|  | 9 | 34/M | 6 | 3 | 15 | 0.5 |

***Note 2. MER signals and spike analysis***


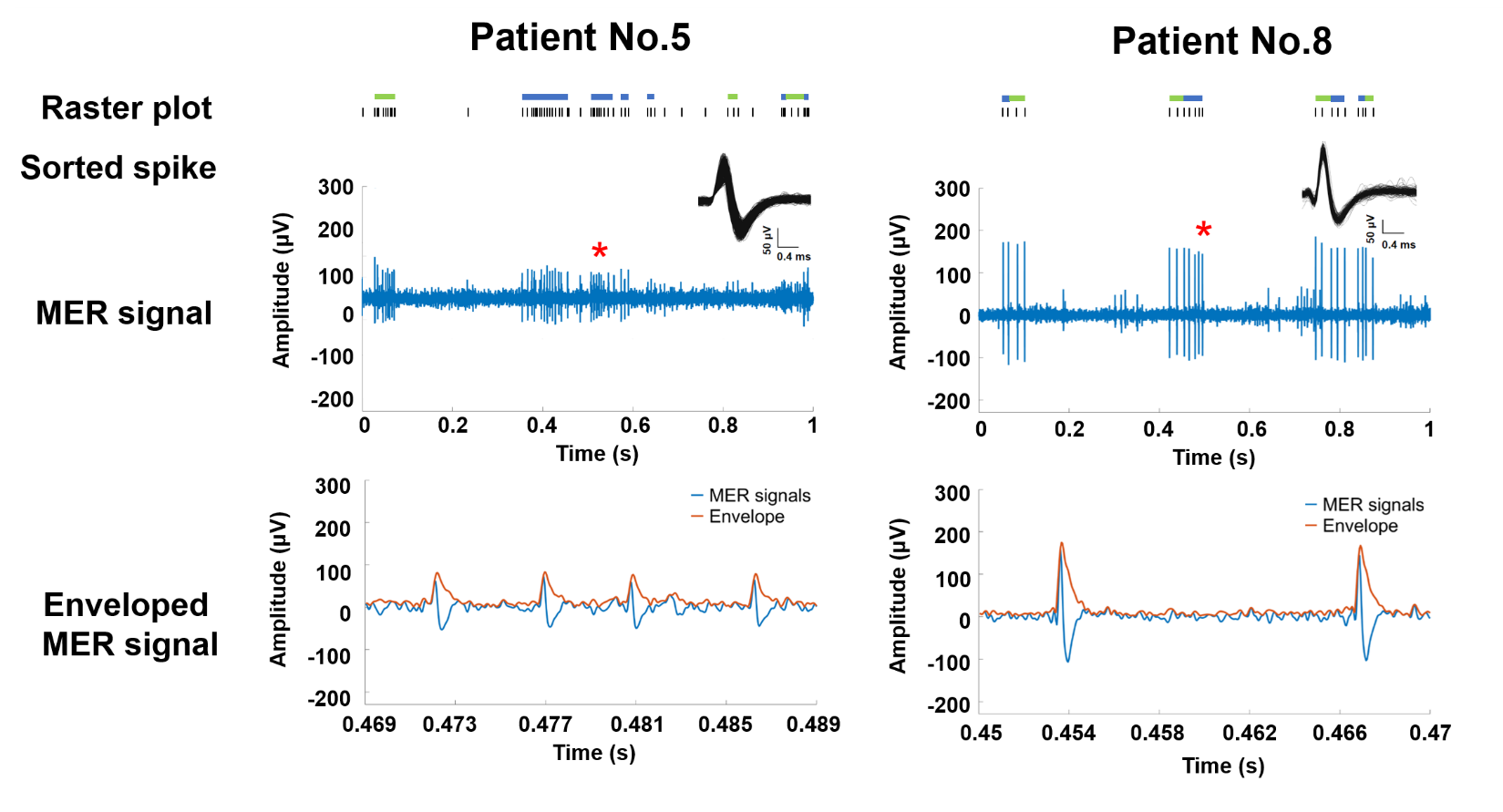


**Figure S1**. **MER signals and spike analysis under GA**. Middle row: two representative examples of single unit activity of MER from the patients No. 5 and No. 8 under GA and their corresponding sorted spikes with congruent spike shape. Top row: a raster plot indicating times of burst firing (ISI < 10 ms, blue rods) and irregular activity (ISI > 10 ms, green rods). Bottom row: the enlarged view of MER signals within the marked area (*) and the corresponding envelope waveforms computed from Hilbert transformed MER signals.


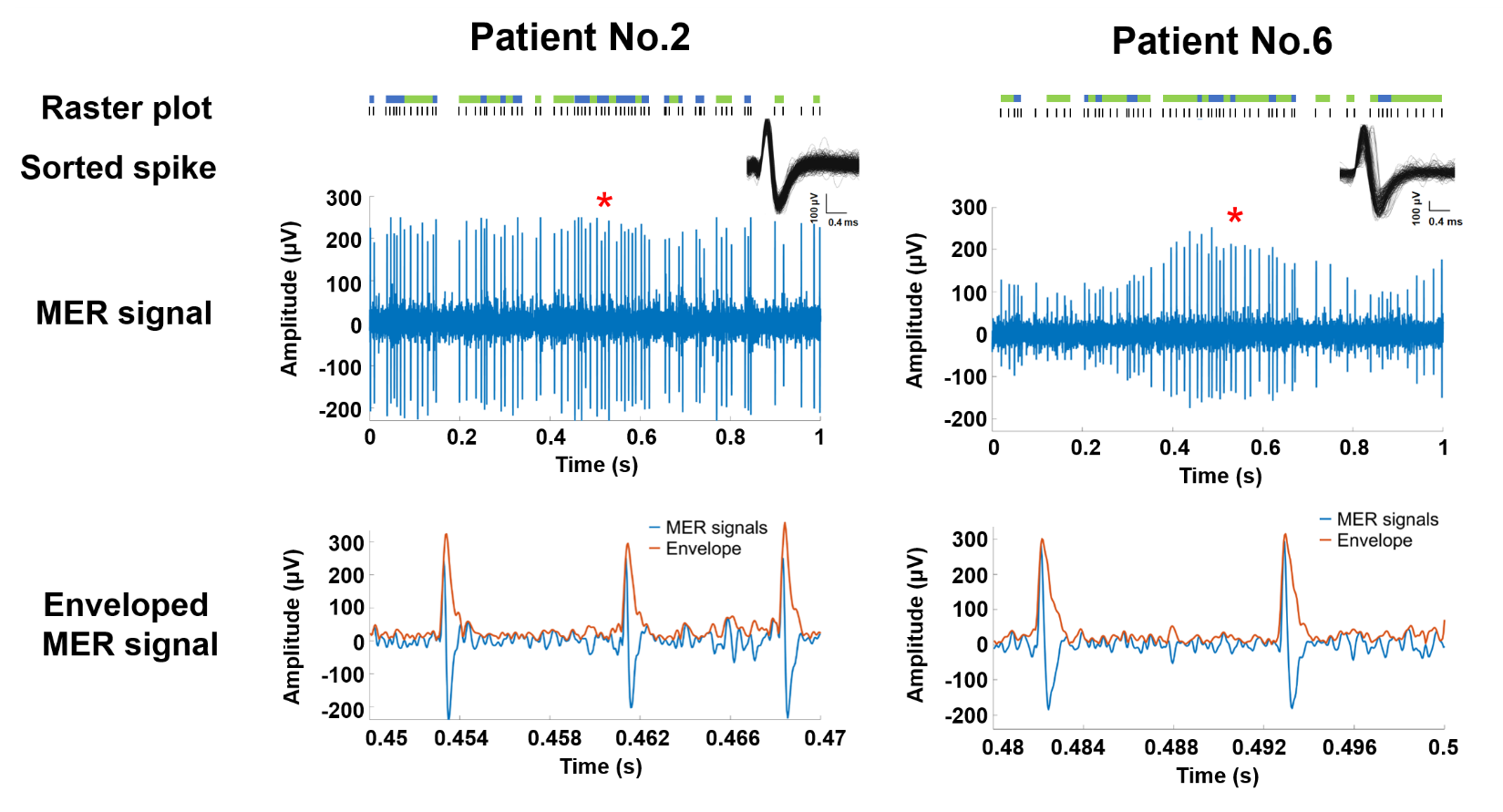


**Figure S2**. **MER signals and spike analysis under LA**. Middle row: representative two examples of single unit activity of MER from the patients No. 2 and No. 6 under LA and their corresponding sorted spikes with congruent spike shape. Top row: a raster plot indicating times of burst firing (ISI < 10 ms, blue rods) and irregular activity (ISI > 10 ms, green rods). Bottom row: the enlarged view of MER signals within the marked area (*) and the corresponding envelope waveforms computed from Hilbert transformed MER signals.
