# Supplementary figures and images for: Decreased Power but Preserved Bursting Features of Subthalamic Neuronal Signals in Advanced Parkinson's Patients under Controlled Desflurane Inhalation Anesthesia
Source: Front Neurosci. 2017 Dec 12;11:701. doi: 10.3389/fnins.2017.00701 (PMC5733027; doi:10.3389/fnins.2017.00701)

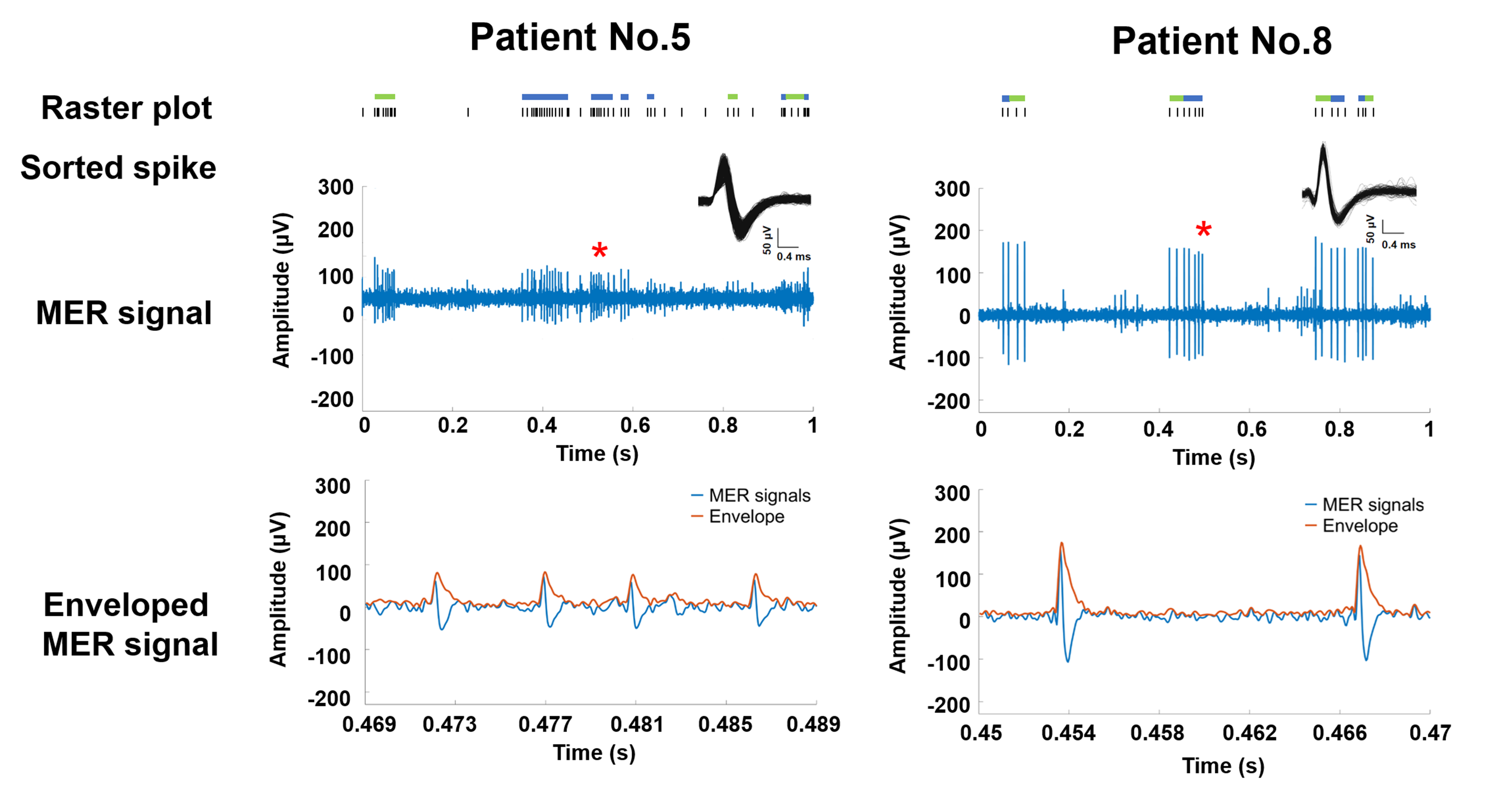

Supplement: Supplementary file 2 [file Image1.TIF]

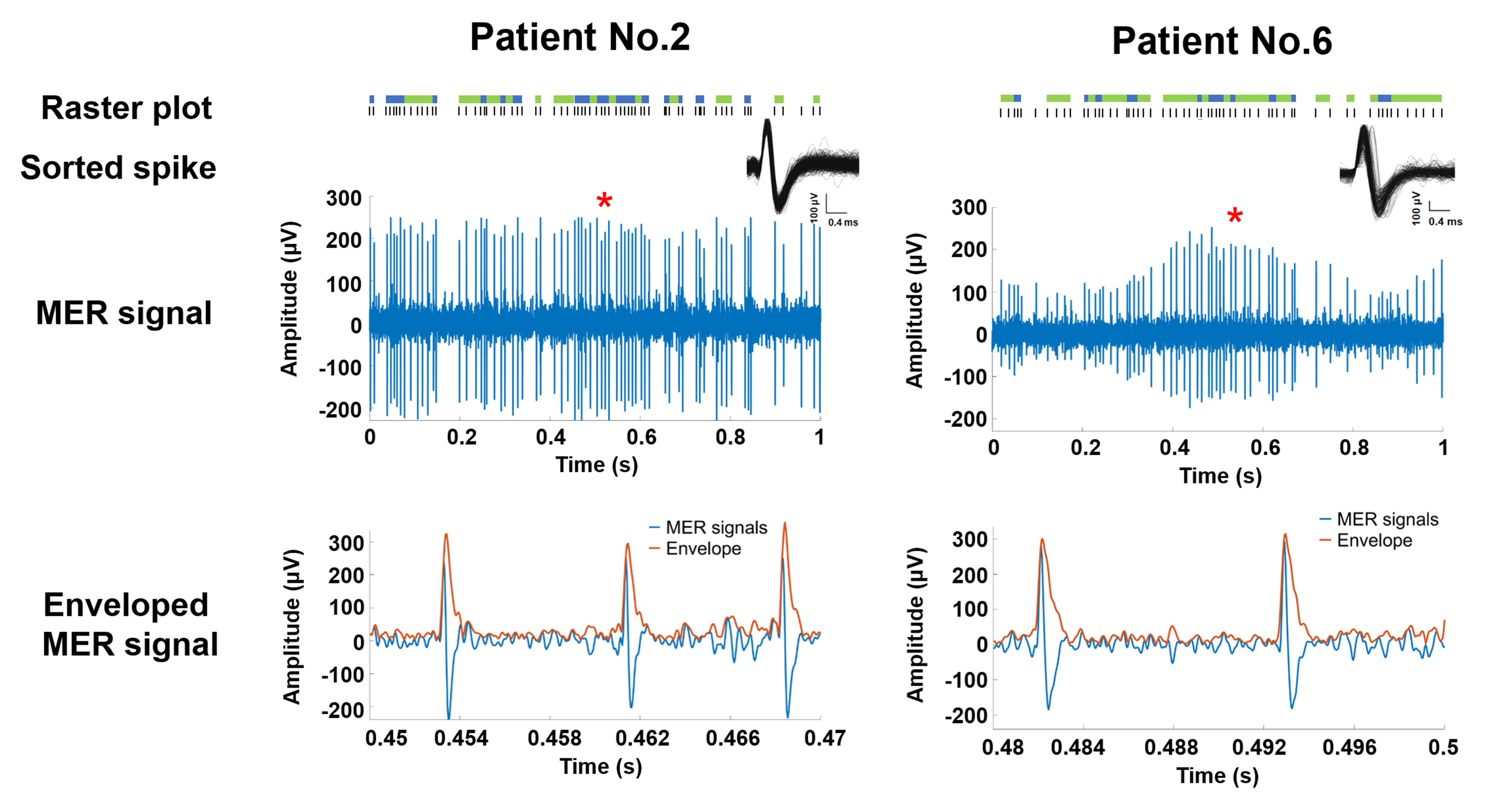

Supplement: Supplementary file 3 [file Image2.TIF]
